# Supplementary material for: Systematic lncRNA mapping to genome-wide co-essential modules uncovers cancer dependency on uncharacterized lncRNAs
Source: eLife. 2022 Jun 13;11:e77357. doi: 10.7554/eLife.77357 (PMC9191893; doi:10.7554/eLife.77357)
Supplement: Figure 6—source data 2. [file elife-77357-fig6-data2.pdf]

Mitra et al.

Figure 6-source data 2: Western blots for Figure 6C

A549

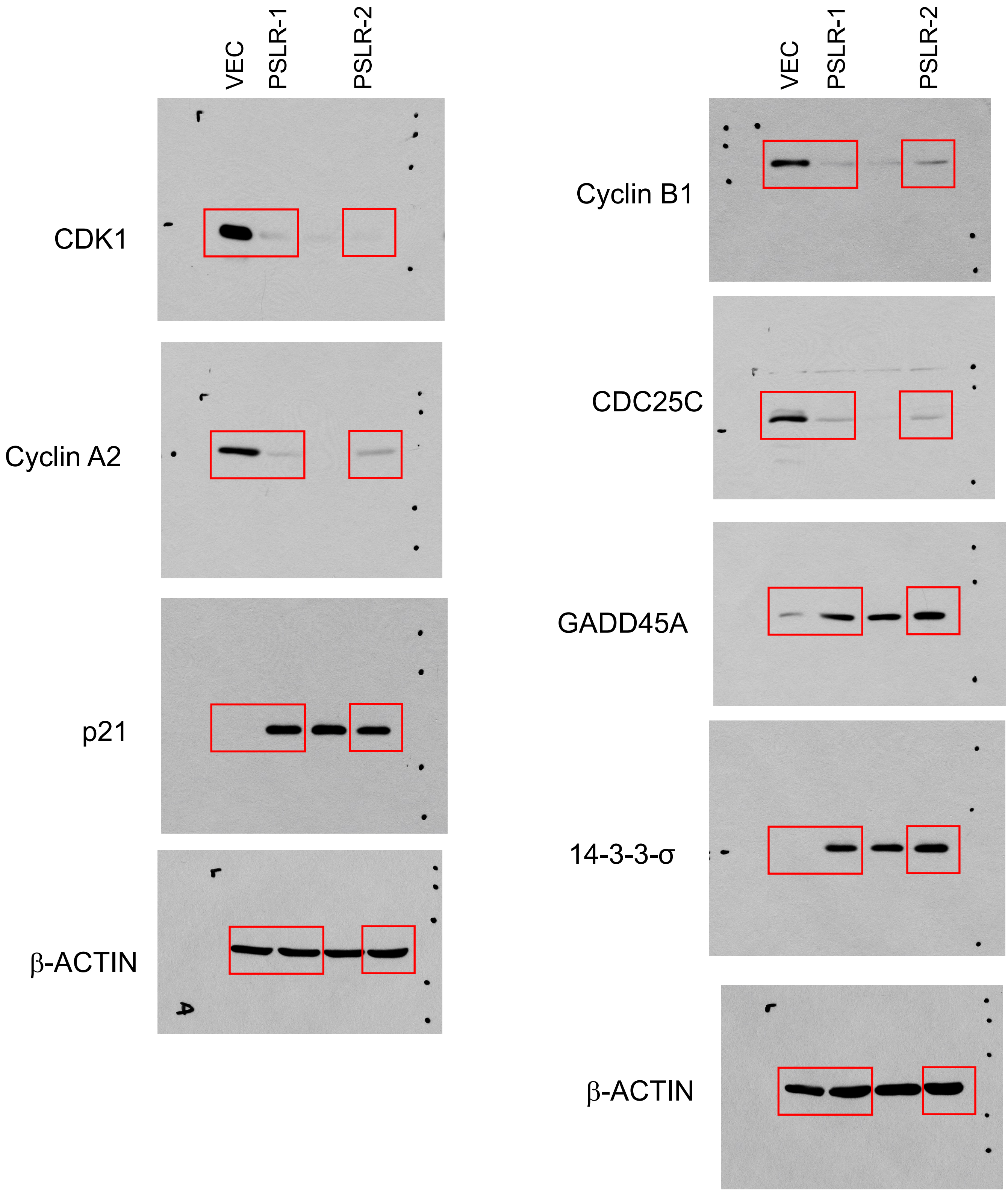

Red boxes indicate lanes used in figure in manuscript

Figure 6-source data 2: Western blots for Figure 6C

Mitra et al.

H460

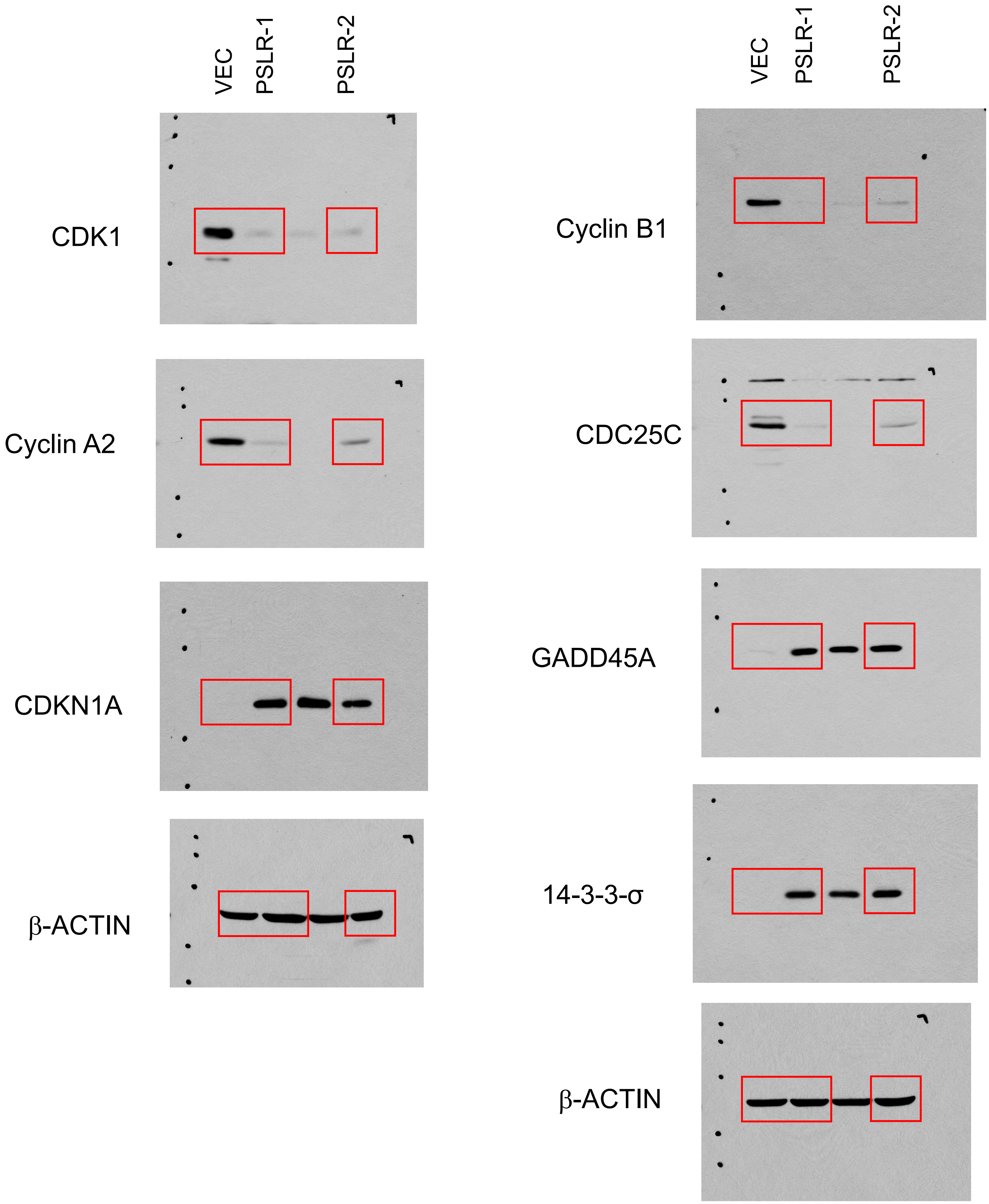

Red boxes indicate lanes used in figure in manuscript
