# Supplementary material for: Systematic lncRNA mapping to genome-wide co-essential modules uncovers cancer dependency on uncharacterized lncRNAs
Source: eLife. 2022 Jun 13;11:e77357. doi: 10.7554/eLife.77357 (PMC9191893; doi:10.7554/eLife.77357)
Supplement: Supplementary file 2. — (a) Differential distribution of expression changes of proliferation inducing and suppressing long noncoding RNAs (lncRNAs) across TCGA cancer types. (b) Summary of the 14 RNA-seq datasets evaluated. (c) Summary of the 17 ChIP-seq datasets evaluated. (d) Primers used for qRT-PCR. [file elife-77357-supp2.docx]

**Supplementary Tables**

**Supplementary file 2a: Differential distribution of expression changes of proliferation inducing and suppressing lncRNAs across TCGA cancer types.**

| **Cancer type** | ***P*-value*** | **FDR*** |
| --- | --- | --- |
| BRCA | 5.86E-16 | 5.86E-15 |
| STAD | 1.12E-14 | 5.61E-14 |
| BLCA | 1.43E-10 | 4.76E-10 |
| HNSC | 1.46E-09 | 3.64E-09 |
| SKCM | 6.66E-09 | 1.33E-08 |
| LUAD | 3.08E-08 | 5.14E-08 |
| UCEC | 9.10E-08 | 1.30E-07 |
| OV | 1.34E-06 | 1.67E-06 |
| LIHC | 0.019822 | 0.022024 |
| KIRC | 0.644311 | 0.644311 |

*Statistical significance was denoted by *P*-value and FDR

**Supplementary file 2b. Summary of the 14 RNA-seq datasets evaluated.**

| **GEO ID** | **Experimental design (*n*)** | **Cell line** | **Pubmed ID** |
| --- | --- | --- | --- |
| GSE111009 | DMSO (3) vs Nutlin (3) | Foreskin fibroblast | 31113863 |
| GSE111009 | DMSO (3) vs Nutlin (3) | MCF10A | 31113863 |
| GSE124508 | DMSO (3) vs Idasanutlin/RG7388^*^ (3) | TTC642 | 30755442 |
| GSE128191 | DMSO (3) vs Nutlin (3) | Neural crest cells | 31178404 |
| GSE47042 | Unstimulated (2) vs Nutlin (2) | MCF7 | 25058159 |
| GSE80716 | DMSO (3) vs Nutlin (3) | MCF7 | 27183917 |
| GSE87668 | DMSO (3) vs Nutlin (3) | U2OS | 27829155 |
| GSE110387 | Untreated (3) vs Ionizing radiation (6) | U2OS | 29476964; 30419821 |
| GSE55727 | Untreated (2) vs Doxorubicin (2) | GM06170 | 25883152 |
| GSE55727 | Untreated (2) vs Doxorubicin (2) | GM00011 | 25883152 |
| GSE79249 | Untreated (2) vs Doxorubicin (2) | HCT116 | 28877474 |
| GSE78512 | Untreated (3) vs 5-Flourouracil (3) at 24 hr | MCF7 | 27602759 |
| GSE78512 | Untreated (3) vs 5-Flourouracil (3) at 48 hr | MCF7 | 27602759 |
| GSE89807 | DMSO (2) vs 5-Flourouracil (2) | SJSA | 28416637 |

^*^Nutlin derivative

**Supplementary file 2c. Summary of the 17 ChIP-seq datasets evaluated.**

| **Treatment group** | **Treatment** | **Cell line** | **Pubmed ID** |
| --- | --- | --- | --- |
| Nutlin | Nutlin | GM12878 | 24120139 |
| Nutlin | Nutlin | IMR90 | 25391375 |
| Nutlin | Nutlin | U2OS | 23775793 |
| Non-Nutlin | 5-Fluorouracil | HCT116 | 25415302 |
| Non-Nutlin | 5-Fluorouracil | IMR90 | 22127205 |
| Non-Nutlin | Actinomycin D | U2OS | 21394211 |
| Non-Nutlin | Actinomycin D | U2OS | 21394211 |
| Non-Nutlin | Cisplatin | Keratinocytes | 24823795 |
| Non-Nutlin | Cisplatin | Keratinocytes | 24823795 |
| Non-Nutlin | Doxorubicin | GM06170, GM00011 | 25883152 |
| Non-Nutlin | Doxorubicin | GM06993, GM11992 | 24120139 |
| Non-Nutlin | Doxorubicin | Keratinocytes | 24823795 |
| Non-Nutlin | Doxorubicin | Keratinocytes | 24823795 |
| Non-Nutlin | Doxorubicin | U2OS | 23775793 |
| Non-Nutlin | Etoposide | U2OS | 21394211 |
| Non-Nutlin | Etoposide | U2OS | 21394211 |
| Non-Nutlin | Etoposide | U2OS | 21394211 |

**Supplementary file 2d. Primers used for qRT-PCR.**

| **Gene** | **Forward Sequence (5'-3')** | **Reverse Sequence (5'-3')** |
| --- | --- | --- |
| *PSLR-1* | AGCCACCCTTGACTGAGGTA | CTCTGTCTTCTGTGCCGTGT |
| *PSLR-2* | GAAAGTCAGCGATTCCTGCG | GTTGGACAGTTCCTCCCTGAG |
| *BAX* | CCCGAGAGGTCTTTTTCCGAG | CCAGCCCATGATGGTTCTGAT |
| *AchR* | AGGACCCTACAGACCCCTCTTC | AGTGTTCATGGTGGCTAGGTG |
| *CDK1* | GCCGGGATCTACCATACCC | AGGAACCCCTTCCTCTTCAC |
| *CCNA2* | GAAAACCATTGGTCCCTCTTG | GGCTGTTTCTTCATGTAACCC |
| *CCNB1* | ATGACATGGTGCACTTTCCTCC | GCCAGGTGCTGCATAACTGG |
| *CDC25C* | CTTCCTTTACCGTCTGTCCAG | CCAAGTTTCCATTGTCATCCC |
| *CDKN1A* | GGAAGACCATGTGGACCTGT | GGATTAGGGCTTCCTCTTGG |
| *GADD45A* | CCCTGATCCAGGCGTTTTG | GATCCATGTAGCGACTTTCCC |
| *SFN* | TGACGACAAGAAGCGCATCAT | GTAGTGGAAGACGGAAAAGTTCA |
| *β-ACTIN* | CACCAACTGGGACGACAT | ACAGCCTGGATAGCAACG |
